# Phenotypic and Genotypic Investigation of Carbapenem-Resistant *Acinetobacter baumannii* in Maharaj Nakhon Si Thammarat Hospital, Thailand

**DOI:** 10.3390/antibiotics12030580

**Published:** 2023-03-15

**Authors:** Sirijan Santajit, Phuangthip Bhoopong, Thida Kong-Ngoen, Witawat Tunyong, Dararat Horpet, Wanfudhla Paehoh-ele, Tasneem Zahedeng, Pornpan Pumirat, Nitat Sookrung, Woranich Hinthong, Nitaya Indrawattana

**Affiliations:** 1 Department of Medical Technology, School of Allied Health Sciences, Walailak University, Nakhon Si Thammarat 80160, Thailand; 2 Research Center in Tropical Pathobiology, Walailak University, Nakhon Si Thammarat 80160, Thailand; 3 Department of Microbiology and Immunology, Faculty of Tropical Medicine, Mahidol University, Bangkok 10400, Thailand; 4 Siriraj Center of Research Excellence in Allergy and Immunology, Faculty of Medicine Siriraj Hospital, Mahidol University, Bangkok 10700, Thailand; 5 Biomedical Research Incubator Unit, Department of Research, Faculty of Medicine Siriraj Hospital, Mahidol University, Bangkok 10700, Thailand; 6 Princess Srisavangavadhana College of Medicine, Chulabhorn Royal Academy, Bangkok 10210, Thailand

**Keywords:** biofilms, CRAB, multidrug resistance, nosocomial infections, REP-PCR

## Abstract

(1) Background: *Acinetobacter baumannii* is well known as a causative agent of severe hospital-acquired infections, especially in intensive care units. The present study characterised the genetic traits of biofilm-forming carbapenem-resistant *A. baumannii* (CRAB) clinical isolates. Additionally, this study determined the prevalence of biofilm-producing *A. baumannii* isolates from a tertiary care hospital and investigated the association of biofilms with the distribution of biofilm-related and antibiotic resistance-associated genotypes. (2) Methods: The 995 non-duplicate *A. baumannii* isolates were identified, and their susceptibilities to different antibiotics were determined using the disk diffusion method. Using the modified microtiter plate assay, the CRAB isolates were investigated for their biofilm formation ability. Hemolysin and protease activities were determined. CRABs were subjected to polymerase chain reaction (PCR) assays targeting *bla*_VIM_, *bla*_NDM_, *bla*_IMP_, *bla*_OXA-23-like_, *bla*_OXA-24-like_, *bla*_OXA-51-like_, *csu*E and *pga*B genes. Individual CRAB isolates were identified for their DNA fingerprint by repetitive element sequence-based (REP)-PCR. (3) Results: Among all *A. baumannii* isolates, 172 CRABs were identified. The major antibiotic resistance gene among the CRAB isolates was *bla*_OXA-51-like_ (100%). Ninety-nine isolates (57.56%) were biofilm producers. The most prevalent biofilm gene was *pga*B (79.65%), followed by *csu*E (76.74%). Evidence of virulence phenotypes revealed that all CRAB exhibited proteolytic activity; however, only four isolates (2.33%) were positive for the hemolytic-producing phenotype. REP-PCR showed that 172 CRAB isolates can be divided into 36-DNA fingerprint patterns. (4) Conclusions: The predominance of biofilm-producing CRAB isolates identified in this study is concerning. The characterisation of risk factors could aid in controlling the continual selection and spreading of the *A. baumannii* phenotype in hospitals, thereby improving patient care quality.

## 1. Introduction

*Acinetobacter baumannii* is a gram-negative coccobacillus responsible for hospital-acquired infections, such as ventilator-related pneumonia, secondary meningitis, skin and soft tissue infections, bacterial septicaemia, urinary tract infections and burn wound infections, particularly in intensive care units (ICUs) [1,2,3]. These infections are often related to a higher mortality rate of up to 26% in hospitalised patients [4]. For ICU patients, the rate ranges from 4% to 43% [5]. *A. baumannii* is the first organism on the World Health Organisation (WHO)’s prioritised list of concerns that pose a significant risk to human health [6]. Recently, the situation has become more severe due to a significant increase in multidrug-resistant (MDR). In addition, *A. baumannii* nosocomial isolates were extensively drug-resistant (XDR) and pandrug-resistant (PDR), some of which were even resistant to tigecycline and colistin, the terminal therapies [1]. Numerous factors have been implicated in the spread of *A. baumannii* as an endemic pathogen throughout the world’s healthcare facilities, including its distinct intrinsic and acquired resistance to several antimicrobial classes, including penicillins, extended spectrum cephalosporins, fluoroquinolones and carbapenems [2], as well as its ability to form a biofilm and persist on biotic and abiotic surfaces, including environmental surfaces and medical equipment [3,7]. Moreover, they can uptake bacterial genetic elements to survive under harsh conditions and antibiotic treatment [8].

Carbapenemase production is the most concerning mechanism in the evolution of carbapenem resistance in *A. baumannii*. They belong to three of the four β-lactamase groups, A, B and D [9]. The OXA β-lactamases are Class D carbapenemases, which are further classified into multiple subgroups, primarily encoded by *bla*_OXA-23-like_, *bla*_OXA-24-like_, *bla*_OXA-58_, *bla*_OXA-48-like_, *bla*_OXA-51-like_ and *bla*_OXA-143-like_ [9,10]. The major OXA type β-lactamase in *Acinetobacter* species is *bla*_OXA-51-like_, chromosomally encoded and unique to these species but may confer carbapenem-resistance when its expression is up-regulated via genetic reorganisation [11]. The metallo-beta-lactamases (MBLs) belong to Class B carbapenemases, most notably the New Delhi carbapenemase *bla*_NDM_ [12]. Moreover, the ability of *A. baumannii* to live on abiotic surfaces, such as catheters and endotracheal tubes, poses a significant barrier to infection control. The capacity to form biofilms is a major factor influencing *A. baumannii*’s capability to thrive in harsh circumstances, making it a key virulence factor [13]. Compared to non-MDR *A. baumannii* (5–24%), MDR *A. baumannii* has a significantly higher rate of biofilm production (80–91%) [14]. Additionally, several investigations have emphasised the function of biofilms in defending *A. baumannii* against the host immune defence [15]. As a result, biofilm-forming bacteria may cause problematic infections. Owing to the capacity of *A. baumannii* to produce a biofilm the bacteria can thrive, adhere to mucosal surfaces, maintain dormancy in deep biofilm layers and persist in a hospital environment under stress conditions [1,8,16]. Several virulence-associated elements are involved in *A. baumannii* biofilm formation, including the two-component system (BfmS/BfmR), chaperon-usher pilus (Csu) encoded by the *csu* operon, the outer membrane protein A (OmpA) expressed by the *omp*A gene, the biofilm-associated protein (Bap) encoded by the *bap* gene, poly-β-(1,6)-*N*-acetyl glucosamine (PNAG), the biosynthesis of extracellular exopolysaccharide (EPS) encoded by *pga*B and the quorum sensing system [17].

By providing insight into the prospective relationship between *A. baumannii* clinical isolates, biofilm production, virulence traits and antibiotic resistance characteristics may help enhance infection control practices in healthcare institutions. Although earlier clinical and epidemiological studies thoroughly investigated the relationship between virulence, biofilm production and antibiotic resistance gene associations and some reports included whole-genome sequencing, the studies in Thailand are still negligible and contradictory [3,16,17,18,19,20,21,22]. Thus, the present study evaluated the occurrence of carbapenem-resistant *A. baumannii* (CRAB) isolates. The biofilm formation capacity of these clinically significant strains was investigated, as were their biofilm-associated genes, *csuE* and *pga*B, drug resistance-related genes, such as *bla*_VIM_, *bla*_NDM_, *bla*_IMP_, *bla*_OXA-23-like_, *bla*_OXA-24-like_ and *bla*_OXA-51-like_ and molecular typing based on repetitive element sequence-based-polymerase chain reaction (REP-PCR) in CRAB clinical isolates. The virulence feature analyses entailed the hemolysis and protease activities.

## 2. Results

### 2.1. Distribution of CRAB Isolates and Antimicrobial Profiles

**Figure 1:**
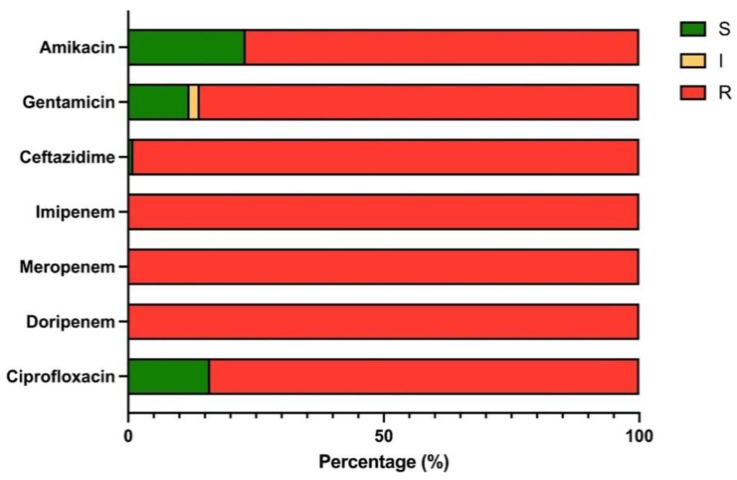
Antimicrobial susceptibility profiles of 172 carbapenem-resistant *A. baumannii* isolates in clinical samples are represented as a percentage. S: sensitive; I: intermediate; R: resistance.

From 995 *A. baumannii* non-duplicate isolates, the highest resistance rates were observed against meropenem (100%), imipenem (100%), doripenem (100%), ceftazidime (99.42%), gentamicin (85%), ciprofloxacin (83.40%) and amikacin (76.7%), respectively [Figure 1, Table 1]. The analysis for resistance to different antibiotic classes revealed that 100% of the isolates were MDR. Among them, 172 isolates (17.29%) were CRAB.

### 2.2. Hemolysis and Protease Activities

In this study, only 4/172 (2.33%) CRAB isolates were positive for hemolysis activity, and all of them have a beta-hemolysis phenotype. 

The primary proteolytic screening results showed that all isolates were positive for protease production based on the formation of the halos zone of hydrolysis around the reaction colonies.

### 2.3. Biofilm Formation Phenotypes of CRAB

**Figure 2:**
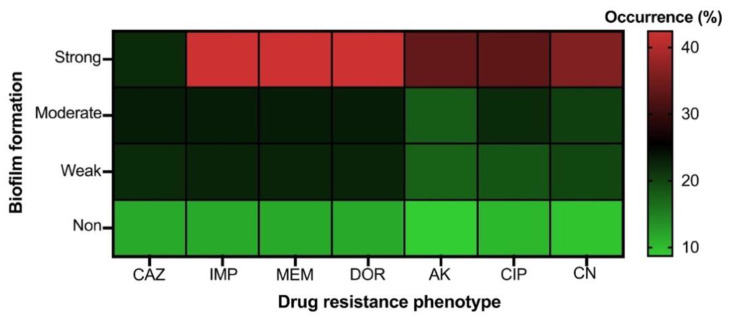
A heatmap demonstrating the percent distribution of 172 carbapenem-resistant *A. baumannii* isolates from nosocomial biofilm-forming and antimicrobial-resistant samples. The color band depicts the percentage of biofilm features associated with a particular drug-resistant phenotype. GraphPad Prism 9 was used to generate the heatmap (La Jolla, CA, USA). CAZ, ceftazidime; IPM, imipenem; MEM, meropenem; DOR, doripenem; AK, amikacin; CIP, ciprofloxacin; CN, gentamicin.

Among all CRAB isolates examined for biofilm formation, 99 (57.56%) were biofilm producers and 73 (42.44%) were non-biofilm producers. The CRAB biofilm-producing strains were divided into three categories: 40 (23.26%) were weak biofilm producers, 39 (22.67%) were moderate biofilm producers and 20 (11.63%) were strong biofilm producers. Heatmap analysis demonstrated differences in the antibiotic resistance profiles of CRAB clinical isolates, which varied in biofilm formation capacity. In Carbapenem-resistant groups (IMP, MEM and DOR), there are a high number of isolates showing strong biofilm formation [Figure 2].

### 2.4. Prevalence of Drug Resistance, Biofilm-Related Genotypes and Genetic Diversity of CRAB

In this study, CRAB isolates harboured the drug resistance-associated genes, including the *bla*_OXA51-like_ gene, at a rate of 100% (172 isolates) and were the most prevalent genotype. In contrast, the *bla*_VIM_, *bla*_IMP_, *bla*_NDM_ and *bla*_OXA23-like_ genes were present in 137 isolates (79.65%), one isolate (0.58%), 21 isolates (12.21%) and 161 isolates (93.60%), respectively. The *bla*_OXA24-like_ gene was not found in CRAB isolates. 

**Figure 3:**
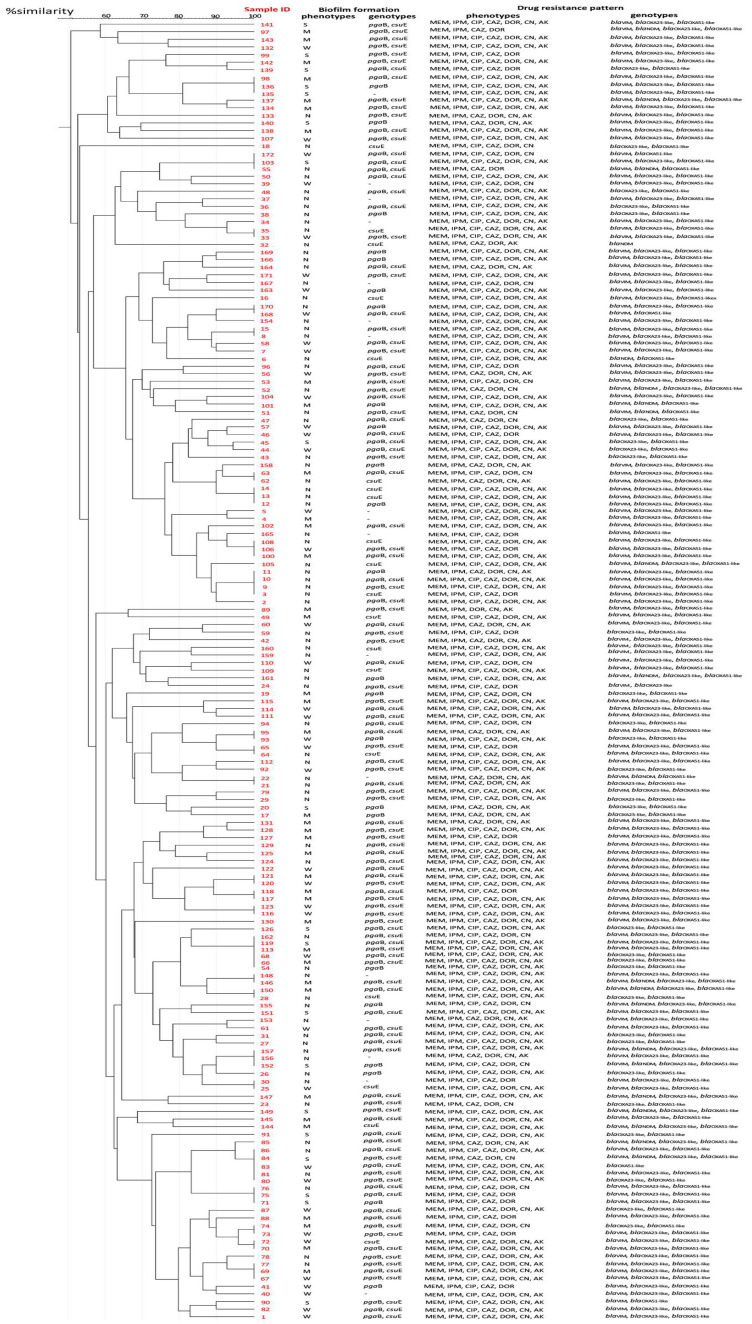
Cluster analysis of repetitive element sequence-based-polymerase chain reaction fingerprints of carbapenem-resistant *A. baumannii* isolates. The dendrogram was generated using GelJ cluster analysis software and the Unweighted Pair Group Method with Arithmetic Mean.

The PCR experiments showed positive results for biofilm-related genes in only the *csu*E gene in 19 (11.05%) isolates, only the *pga*B gene in 24 (13.395%) isolates and both the *csu*E and *pga*B genes in 113 (65.70%) isolates. However, there were 16 (9.30%) isolates that did not carry both genes. Our findings revealed a high prevalence of biofilm-forming and biofilm-related genes (*csu*E and *pga*B) in CRAB strains in the study region [Table 2]. Moreover, Figure 3 depicts an unweighted pair group method with an arithmetic mean dendrogram derived from REP-PCR of 172 CRAB strains. The REP-PCR cluster for bacteria with at least 70% coefficient similarity generated 36 clonal diversities.

## 3. Discussion

Recently, the role of *A. baumannii* in nosocomial infections, its notable ability to develop antimicrobial resistance and its involvement in severe clinical infections have raised considerable attention [23]. Treating these bacteria, particularly MDR and broad-spectrum beta-lactamase strains, is of critical importance [24]. Carbapenems are currently the treatment of choice for MDR *A. baumannii* infections that are resistant to third generation cephalosporins. However, the number of carbapenem-resistant strains is rising [25]. As a result of the large prevalence rate of this infection and the varying patterns of antibiotic resistance in various geographical locations, the surveillance of the prevalence rate and antibiogram in different parts of the world is crucial. These data would help determine the distribution of resistance patterns and select the most suitable medication regimen [26].

In our investigation, 172 of the 995 *A. baumannii* isolates from clinical samples were carbapenem-resistant. Moreover, all CRAB isolates were MDR (100%). The resistance to the cephalosporin antibiotics, gentamicin and ciprofloxacin, was more than 80%. All isolates were resistant to imipenem, meropenem and doripenem. The antibiotic resistance in the present study was similar to the reports from Iran [27]. Almost all the isolates in our study carried the *bla*_OXA-51-like_ genes. The intrinsic *bla*_OXA-51-like_ gene’s identification of the *A. baumannii* isolates provided proof of their identity. The *bla*_OXA-51-like_ genes are an easy and robust tool for identifying *A. baumannii* [28,29,30]. These carbapenem-resistant isolates have been attributed to a strong promoter-driven upregulation of the *bla*_OXA51-like_ genes when related to the *ISAba1* gene 7 bp upstream, as previously reported [31]. The *bla*_OXA-51-like_ gene was the most frequently detected carbapenemase gene among all clinical CRAB isolates (100%) in the current study, and it is also commonly found in health care facilities worldwide, followed by the *bla*_OXA-23-like_, *bla*_VIM_, *bla*_NDM_ and *bla*_IMP_ genes at 93.60%, 79.65%, 12.21% and 0.58%, respectively. Furthermore, the spread of CRAB carrying carbapenem-resistance genes was proven in many reports from other countries [26,28,32,33,34]. Additionally, none of the isolates in this study possessed the *bla*_OXA-24-like_ gene, which is consistent with others [35,36,37,38]. The finding is presumed to correlate with clonal expansion [37,38,39]. Moreover, the resistance to carbapenems in these strains may be clarified by the participation of other mechanisms of resistance to carbapenem, such as the modified permeability or additional carbapenemase enzymes not examined in this study. Examples of non-carbapenemase carbapenem-resistance mechanisms in *A. baumannii* include reduced membrane porin density [40], decreased drug affinity caused by PBP downregulation [41] and efflux pump (EP) mechanisms.

*A. baumannii*’s pathogenicity and resistance to unfavourable environmental conditions correlate with numerous virulence factors, such as the capacity to generate hemolysin, lipase, lecithinase and protease, as well as the ability to form biofilms and quorum sensing [42,43]. The growth behaviour of bacteria in biofilms is altered, reducing their susceptibility to specific antimicrobial treatments [44]. Along with known traditional drug-resistant mechanisms, alternative strategies contribute to the resilience of bacteria in biofilms, such as slow or partial permeation of antimicrobial drugs into the biofilm, a unique microenvironment in the biofilm and altered growth behaviour of microbes within biofilms. Since biofilms are multicellular, these mechanisms result in bacterial resistance and unsuccessful treatment efforts [45]. Approximately 57.56% of the CRAB isolates in this study were biofilm-forming strains. According to previous studies, biofilm-forming bacteria have a substantially longer life than those that do not form biofilms (36 versus 15 days, *p*-value < 0.001) [46,47]. The capacity of *A. baumannii* to form biofilms improved colonisation and persistence, allowing for higher rates of nosocomial infections, particularly device-associated illnesses [48]. Several factors, including environmental factors and numerous cell signals, influence *A. baumannii* biofilm development by influencing signalling, cell-to-cell interaction and scaffolding functions [49]. Furthermore, the genes associated with biofilms offer a comprehensive perspective on surface adhesion and biofilm development. Among these genes were *pga*B and *csu*E [50]. The Csu chaperone-usher pili assembly system is regulated by the BfmS/BfmR two-component system (*pga*B), and pgaABCD is responsible for producing poly-1,6-*N*-acetylglucosamine [51]. The initial surface attachment phase of biofilm formation is mediated by pili, composed of proteins encoded by the *csu* operon. Previous research demonstrated that BfmR stabilised the transcription of the *csu* operon genes, which are vital in biofilm formation [52,53]. The presence of *pga*B and *csu*E as biofilm encoding genes in CRAB isolates from admitted patients was studied. Our results revealed that *pga*B and *csu*E were present in most isolates similar to those reported by Zeighami et al. [3] who reported these genes in *A. baumannii* recovered from ICU patients. In this study, the vital virulence activities of CRAB illustrated the evidence of biofilm production, hemolysis and protease activities. This finding is consistent with a previous study by Dahdouh and Hajjar [54], which revealed that the isolates produced biofilm, caused blood hemolysis in an agar plates and exhibited proteolytic activity. 

PCR-based fingerprinting methods, such as REP-PCR, are easy, quick and low-cost, with strong discriminatory power for identifying *A. baumannii*. The results of REP-PCR can be achieved in a reasonably short amount of time, as demonstrated by a review by Sabat et al. This is also the reason this procedure is less expensive. REP-PCR is highly discriminating for several bacterial species [55]. Previous research found that the REP-PCR discrimination power was adequate and correlated with PFGE [56,57]. REP-PCR was helpful in the epidemiological analysis of hospital epidemics. Many investigations employed PCR-based fingerprinting to identify *A. baumannii* clinical isolates [3,58]. By typing *A. baumannii* with REP-PCR, the obtained patterns were classified as distinct REP-PCR clusters that provided evidence of possible clonal expansion among the different isolates. Despite several reports of clonality in the literature, it is possible to identify the levels of clonal diversity among the CRAB strains. 

However, the underlying molecular reason for the bacterium’s rising prevalence and antibiotic resistance still needs to be fully understood. Molecular detection and whole-genome sequencing should be employed to better understand drug resistance and bacterial pathogenesis mechanisms. Furthermore, integrating molecular, genomic and bioinformatics tools resulted in genomic epidemiology approaches, which gradually increased in various fields of pathogen surveillance and developed control strategies.

## 4. Materials and Methods

### 4.1. Study Settings and Ethical Approval 

The non-duplicate 995 *A. baumannii* isolates were attained from February to September 2021 at the Maharaj Nakhon Si Thammarat Hospital. The study was approved by the Human Research Ethics Committee of Walailak University (protocol number: WUEC-21-027-01). 

### 4.2. Bacterial Isolation and Identification 

The stored isolates were recovered and confirmed as *A. baumannii* by conventional microbiological methods such as gram stain, gram-negative coccobacilli; the oxidase test, negative; triple sugar iron test, K/N; Simmons citrate agar, positive; motility-indole-lysine, negative-negative-positive; OF-maltose, non-oxidiser; OF-glucose, oxidiser and growth on MacConkey agar at 42 °C [59]. 

### 4.3. Antimicrobial Susceptibility Testing

The Kirby–Bauer disc diffusion method was used to determine the antimicrobial susceptibility of all 995 *A. baumannii* clinical isolates in accordance with the instructions of the Clinical and Laboratory Standards Institute (CLSI) 2020 [60]. The following antibiotics were tested: ceftazidime (CAZ, 10 μg), imipenem (IMP, 10 μg), meropenem (MEM, 10 μg), amikacin (AK, 30 μg), ciprofloxacin (CIP, 5 μg), doripenem (DOR, 10 μg) and gentamicin (CN, 10 μg). MDR was defined as acquired resistance to more than three classes of antibiotics [61].

### 4.4. Hemolysis Assay and Protease Activity 

The phenotypic hemolysin activity was determined using the streaking and spot methods on a blood agar plate assay, as described previously [62]. All plates were incubated for 24 h at 37 °C. On blood agar plates, the hemolysis was visualised. The presence of a zone that is lightly cleared in the media and around bacterial colonies when using the streaking method indicated that CRAB could haemolyse red blood cells. Based on the appearance of a greyish-greenish colony encircled by a clear zone, beta-hemolysis was detectable using the spot method. Additionally, the skim milk plate method tested all isolates for proteolytic activity. The inoculated plates were incubated for 4 h at 37 °C. A clear zone surrounding a bacterial colony indicated a positive outcome. The experiment was carried out in triplicate.

### 4.5. Biofilm Formation Using Microtiter Plate Assay

The microtiter plate technique assessed the biofilm-forming capacity of CRAB clinical isolates. The isolates were cultivated overnight at 37 °C in tryptic soy broth (TSB) (Oxoid, Basingstoke, UK) and adjusted to No. 0.5 McFarland standards. A 96-well flat-base plate was inoculated with 20 microliters of fresh bacterial culture and incubated for 24 h at 37 °C in 180 microliters of TSB supplemented with 0.25% (*w*/*v*) glucose. The plates were rinsed thrice with phosphate-buffered saline after incubation. Crystal violet (1%; *v*/*v*) was used to stain the attached cells for 20 min. The stained dye of the adhering cells was dissolved with absolute ethanol. The solution’s optical density was measured at 580 nm [63]. This absorbance value of the solution indicated the biofilm-forming capacity of the isolate. As a negative control, sterile TSB supplemented with glucose was used. The average reading was achieved with three replicated experiments. The term ‘ODc’ was defined as the mean optical density (OD) of the negative control plus three standard deviations (cut-off OD). The respective biofilm formation degrees of the CRAB isolates were reported as follows: strong biofilm formation (4 × ODc < OD), moderate biofilm formation (2 × < ODc < OD 4 × ODc), weak biofilm formation (ODc < OD < 2 × ODc) and non-biofilm formation (OD < ODc) [16].

### 4.6. Genotypic Characterisation of Antimicrobial Resistance and Biofilm-Associated Genes by Polymerase Chain Reaction (PCR)

The CRAB isolates were cultivated in Luria-Bertani broth at 37 °C. The DNA template for the PCR experiment was prepared using a genomic DNA extraction kit (Geneaid, Taiwan). Briefly, the bacterial pellet was collected from 1 mL of an overnight bacterial culture by centrifuging for 1 min at 14,000× *g*. Then, the pellet was resuspended in 180 μL of GT buffer and mixed by vortex. The suspension was added to 20 μL of Proteinase K and incubated at 60 °C for 10 min. The sample was added with 200 μL of GB Buffer, mixed and incubated at 70 °C for 10 min. Two-hundred microliters of absolute ethanol were added into the sample and mixed well. The sample solution was transferred into a GD Column, then centrifuged at 14,000× *g* for 2 min. The sample was washed with washing buffers. The extracted DNA was eluted from the column by elution buffer and determined the DNA quantity using a NanoDrop-1000 spectrophotometer (Thermo Fisher Scientific, Wilmington, NC, USA). 

PCR was used to detect the presence of drug resistance-associated genes, including *bla*_VIM_, *bla*_IMP_, *bla*_NDM_, *bla*_OXA-23-like_, *bla*_OXA-24-like_ and *bla*_OXA-51-like_ genes and biofilm-related genes, such as *csu*E and *pga*B genes. The primer sets, annealing temperature and sizes of the expected amplicons are demonstrated in Table S1. The amplification reaction (25 μL) contains *Taq* DNA polymerase, 10× *Taq* buffer, 25 mM MgCl_2_, 10 mM dNTPs, 10 mM of each forward and reverse primer solution, 1.25 units/L (Thermo Scientific, Wilmington, NC, USA) and 100 ng/L of DNA template. The thermal cycles were carried out with the initial denaturation at 95 °C for 5 min, followed by 35 cycles of denaturing at 95 °C for 30 s, annealing at a temperature specific for each primer pair for 30 s and extension at 72 °C for 30 s, with a final extension at 72 °C for 7 min. The PCR amplicon was separated on a 1% (*w*/*v*) agarose gel, stained with the nucleic acid staining dye, SafeView^TM^ FireRed (ABM Good, Richmond, BC, Canada), and examined under UV transillumination using the ChemiDoc MP imaging system (Bio-Rad, Hercules, CA, USA). Subsequently, the purified PCR amplicons were sequenced (Macrogen, Seoul, Republic of Korea). The NCBI web-based genome analysis, including the DDBJ/EMBL/GenBank databases, was used for sequence analysis to confirm the target amplicons.

### 4.7. Molecular Typing and Clonal Relationship between CRAB Strains by Repetitive Element Sequence-Based PCR (REP-PCR)

To distinguish between bacterial strains, DNA fingerprinting was investigated by using REP-PCR, a molecular typing method. The REP-like elements in the genomic DNA isolated from CRAB isolates were amplified using the primer pairs REP1 (5′-IIIGCGCCGICATCAGGC-3′) and REP2 (5′-ACGTCTTATCAGGCCTAC-3′), as previously described [6]. Briefly, a 25 µL reaction mixture comprising 100 ng of chromosomal DNA, 2.5 µL of 10× *Taq* buffer, 0.5 µL of 10 mM dNTP mix, 1.5 U of *Taq* DNA polymerase (Thermo Fisher Scientific, Wilmington, NC, USA), 50 pmoL of each primer and 1.25 µL of dimethyl sulfoxide was added. The amplification procedure included initial denaturation at 94 °C for 10 min, followed by 30 cycles of denaturation (94 °C, 1 min), annealing (40 °C, 1 min), extension (72 °C, 2 min) and a single final extension (72 °C, 16 min). The PCR products were electrophoresed in a 1.2% (*w*/*v*) agarose gel. The lanes and bands were determined against a 100 bp plus ladder lane (Thermo Fisher Scientific, Wilmington, NC, USA). 

For determining genotyping and clonal relationships between CRAB strains, the gels were stained and the DNA bands were photographed and visualised using a UV transilluminator. The images of DNA banding patterns were analysed with GelJ software version 3.0 (San Diego, CA, USA) and the dendrogram was created using the unweighted pair group method with arithmetic averages and Dice’s similarity coefficient with a tolerance of 1.0% [64,65]. A similarity of 70% or greater indicated the same REP-PCR genotype, whereas a similarity of less than 70% indicated different REP-PCR genotypes.

### 4.8. Statistical Data Analysis

The data were analysed using GraphPad Prism 9. All analyses were performed using three separate experiments. The prevalence of CRABs, genotypic and phenotypic biofilm formation and drug resistance were demonstrated as percentages. The relationship between biofilm formation and antibiotic susceptibility was demonstrated by heatmap analysis and the chi-squared test where a *p*-value of <0.05 was considered to be statistically significant. 

## 5. Conclusions

The high occurrence of biofilm-producing MDR clinical isolates found in this study is concerning. The rising prevalence of carbapenem resistance is alarming. Our findings highlighted the significance of *csu*E and/or *pga*B genes, carbapenemase-encoding genes and the emergence of drug resistant strains as potential risk factors linked with biofilm-producing *A. baumannii* clinical isolates. These surveillance outcomes that continually monitor the epidemiological and susceptibility profiles of antimicrobials used against disseminating pathogens in healthcare environments might be one of the policies that help treat nosocomial infections caused by bacteria and limit the spread of virulent strains. It could assist health officials and policymakers in managing continuous selection and transfer of this trait within healthcare facilities, thereby improving patient care quality.

## Figures and Tables

**Table 1:** Antibiogram pattern among carbapenem-resistance *A. baumannii* isolates.

Antimicrobial Agents	Antimicrobial Susceptibility Profile of CRAB Isolates (*n* = 172)		
	Susceptible *n* (%)	Intermediate *n* (%)	Resistance *n* (%)
Group Cephalosporins			
ceftazidime (CAZ)	1 (0.58)	0 (0)	171 (99.42)
Group Aminoglycosides			
gentamicin (CN)	21 (12.21)	4 (2.33)	147 (85.47)
amikacin (AK)	40 (23.26)	0 (0)	132 (76.74)
Group Carbapenem			
meropenem (MEM)	0 (0)	0 (0)	172 (100)
imipenem (IPM)	0 (0)	0 (0)	172 (100)
doripenem (DOR)	0 (0)	0 (0)	172 (100)
Group Fluoroquinolones			
ciprofloxacin (CIP)	27 (15.70)	0 (0)	145 (84.30)

*n*, number of isolates.

**Table 2:** Distribution of biofilm formation of the isolates with different antibiotic resistance phenotypes among *A. baumannii* isolates.

Antibiotics	Antibiotic Resistance		Antibiotic Susceptible		*p*-Value
	Biofilm Producers Isolates (%)	Non-Biofilm Producers Isolates (%)	Biofilm Producers Isolates (%)	Non-Biofilm Producers Isolates (%)	
Amikacin	73 (55.30%)	59 (44.70%)	26 (65%)	14 (35%)	0.277
Gentamicin	85 (57.82%)	62 (42.18%)	14 (66.67%)	7 (33.33%)	0.441
Ceftazidime	73 (42.69%)	98 (57.31%)	1 (100%)	0 (0%)	0.248
Imipenem/Meropenem/Doripenem	99 (57.56%)	73 (42.44%)	0 (0%)	0 (0%)	NT
Ciprofloxacin	88 (60.69%)	57 (39.31%)	12(44.44%)	15(55.56%)	0.166

NT, not test.

## Data Availability

The data of this study are available from the corresponding author upon request.

## Supplementary Materials

The following supporting information can be downloaded at: https://www.mdpi.com/article/10.3390/antibiotics12030580/s1, Table S1: PCR primers used for amplification of different drug resistance- and biofilm associated genes.
