# Supplementary material for: Phenotypic and Genotypic Investigation of Carbapenem-Resistant Acinetobacter baumannii in Maharaj Nakhon Si Thammarat Hospital, Thailand
Source: Antibiotics (Basel). 2023 Mar 15;12(3):580. doi: 10.3390/antibiotics12030580 (PMC10044629; doi:10.3390/antibiotics12030580)
Supplement: Supplementary file 1 [file antibiotics-12-00580-s001.zip › antibiotics-2258998-SI.pdf]

antibiotics

Supplementary data

Phenotypic and Genotypic Investigation of Carbapenem-Resistant *Acinetobacter baumannii* in Maharaj Nakhon Si Thammarat Hospital, Thailand

Sirijan Santajit [1,2,3], Phuangthip Bhoopong [1], Thida Kong-Ngoen [3], Witawat Tunyong [3], Dararat Horpet [1], Wanfudhla Paehoh-ele [1], Tasneem Zahedeng [1], Pornpan Pumirat [3], Nitat Sookrung [4,5], Woranich Hinthong [6] and Nitaya Indrawattana [3,*]

1 Department of Medical Technology, School of Allied Health Sciences, Walailak University, Nakhon Si Thammarat 80160, Thailand
2 Research Center in Tropical Pathobiology, Walailak University, Nakhon Si Thammarat 80160, Thailand
3 Department of Microbiology and Immunology, Faculty of Tropical Medicine, Mahidol University, Bangkok 10400, Thailand
4 Siriraj Center of Research Excellence in Allergy and Immunology, Faculty of Medicine Siriraj Hospital, Mahidol University, Bangkok 10700, Thailand
5 Biomedical Research Incubator Unit, Department of Research, Faculty of Medicine Siriraj Hospital, Mahidol University, Bangkok 10700, Thailand
6 Princess Srisavangavadhana College of Medicine, Chulabhorn Royal Academy, Bangkok 10210, Thailand
* Correspondence: nitaya.ind@mahidol.ac.th; Tel.: +66-2-354-9100 (ext. 1598)

antibiotics

MDPI

Table. S1 PCR primers used for amplification of different drug resistance- and biofilm associated genes.

Gene Name	Oligonucleotide Sequence (5′-3′)	Product Size (bp)	Annealing Temperature (°C)	References
*bla*VIM	Forward: GATGGTGTTTGGTCGCATA<br>Reverse: CGAATGCGCAGCACCAG	390	60	[23,24]
*bla*IMP	Forward: GAAGGCGTTTATGTTCATAC<br>Reverse: GTAAGTTTCAAGAGTGATGC	382	56	[23,24]
*bla*NDM	Forward: GGTTTGGCGATCTGGTTTTC<br>Reverse: CGGAATGGCTCATCACGATC	621	61	[23,24]
*bla*OXA-23-like	Forward: GATCGGATTGGAGAACCAGA<br>Reverse: ATTTCTGACCGCATTTCCAT	501	60	[24,25]
*bla*OXA-24-like	Forward: GGTTAGTTGGCCCCCTTAAA<br>Reverse: AGTTGAGCGAAAAGGGGATT	246	61	[24,25]
*bla*OXA-51-like	Forward: TAATGCTTTGATCGGCCTTG<br>Reverse: TGGATTGCACTTCATCTTGG	353	60	[24,25]
csuE	Forward: ATGCATGTTCTCTGGACTGATGTTGAC<br>Reverse:<br>CGACTTGTACCGTGACCGTATCTTGATAAG	976	60	[3]
pgaB	Forward: AAGAAAATGCCTGTGCCGACCA<br>Reverse: GCGAGACCTGCAAAGGGCTGAT	490	57	[3]
